# Correlation between Tumour Associated Macrophage (TAM) Infiltration and Mitotic Activity in Canine Soft Tissue Sarcomas †

**DOI:** 10.3390/ani11030684

**Published:** 2021-03-04

**Authors:** Riccardo Finotello, Kate Whybrow, Giulia Scarin, Lorenzo Ressel

**Affiliations:** 1 Department of Small Animal Clinical Science, Institute of Infection, Veterinary and Ecological Science, University of Liverpool, Neston CH64 7TE, UK; 2 Institute of Infection, Veterinary and Ecological Science, University of Liverpool, Neston CH64 7TE, UK; katewhybrow@gmail.com; 3 School of Veterinary Medicine and Science, University of Nottingham, Sutton Bonington, Loughborough LE12 5RD, UK; giulia.scarin@nottingham.ac.uk; 4 Department of Veterinary Anatomy Physiology and Pathology, Institute of Infection, Veterinary and Ecological Science, Neston CH64 7TE, UK; ressel@liverpool.ac.uk

**Keywords:** macrophages, TAM, mitosis, microenvironment, soft tissue sarcoma, dog, canine

## Abstract

**Simple Summary:**

Tumor-associated macrophages (TAMs) are a class of immune cells present in solid tumors and they are involved in cancer-related inflammation. However, to our knowledge, literature about TAMs in canine soft tissue sarcomas (STSs) is limited to absent. Here we analyzed 38 STSs retrieved from the veterinary pathology archive. Only STSs arising from limbs and the trunk were included. Oral, visceral STSs, and tumors mimicking STSs were excluded. TAMs were identified by means of immunohistochemistry and were counted in 10 consecutive tumors areas, where no confounding factors such as necrosis or other inflammatory cells could be identified. Associations between numbers of TAMs and tumor features were investigated. TAMs were evident in all STSs and ranged between 6% to 62% of the cells in the microscopic field. The number of TAMs positively correlated with the STSs’ histologic grade. The present findings suggest that TAMs are present in higher numbers when STSs are of aggressive histological grade and especially in those with a high number of proliferating cancer cells. The abundant presence of TAMs in high grade STSs may also increase the likelihood of a pathologist misdiagnosing STS for tumors where macrophages are the actual cancerous component, such as histiocytic sarcomas.

**Abstract:**

Tumour-associated macrophages (TAMs) are an important part of the tumour microenvironment but knowledge of their distribution in canine soft tissue sarcomas (STSs) is limited to absent. We analysed 38 STSs retrieved from the veterinary pathology archive; oral and visceral STSs, synovial cell sarcoma, tumours of histiocytic origin, haemangiosarcoma, carcinosarcomas, and undifferentiated tumours were excluded. Iba-1 positive, non-neoplastic tumour infiltrating cells (morphologically indicative of macrophages) were classified as TAMs and were counted in 10 consecutive tumours areas, where no necrosis or other inflammatory cells could be identified. Associations between numbers of TAMs and mitoses, differentiation, and necrosis scores or grade were investigated. TAMs were evident in all STSs and ranged between 6% to 62% of the cells in the microscopic field. The number of TAMs positively correlated with the STSs’ histologic grade. When the components of the grade were analysed separately, TAMs were statistically correlated with mitoses, but not with differentiation or necrosis score. The present findings suggest that TAMs are present in higher numbers when STS proliferation is the predominant feature that drives tumour grade. The abundant presence of TAMs in high-grade STSs may also increase the likelihood of a pathologist misdiagnosing STS for histiocytic sarcoma.

## 1. Introduction

Non-resolving inflammation is one of the hallmarks of cancer, and among inflammatory cells, tumor-associated macrophages (TAMs) represent probably the most relevant component of the leucocytes infiltrate [1,2,3]. TAMs are derived from peripheral blood monocytes that have been recruited by chemokines; these can be divided into selectively activated (M2) and classically activated (M1) macrophages [1,3,4]. M2 macrophages are mainly activated by helper T-cell (Th) 2 secreted cytokines (such as interleukin (IL)-4, IL-10, and IL-13) and are thought to play a relevant role in cancer initiation and promotion, immune suppression, metastasis, and angiogenesis [1,3]. Conversely, M1 macrophages are primarily considered as “tumoricidal”, being physiologically involved in helper T-cell (Th) 1 responses to infection, the production of pro-inflammatory cytokines and phagocytize microbes, initiating an immune response and being mainly activated by interferon-γ or lipopolysaccharide [1,3]. 

In human oncology, numerous studies have demonstrated that a high number of infiltrating TAMs with M2 phenotype represent a negative prognostic factor in various tumors: non-gynecologic leiomyosarcomas, gastric cancer, urogenital cancer, head and neck cancer, lymphomas, and cholangiocarcinoma [3,5]. On the contrary, tumor infiltration by macrophages with M1 phenotype seem to favor a better prognosis in non-small cell lung cancer and ovarian cancer [5,6], among others. However, this is not always the case as M2 TAMs have also been associated with lower tumor stages and/or improved survival in tumors such as Ewing sarcoma and colorectal carcinomas [7,8]. Overall, differentiation among TAMs of the M1 and M2 phenotype is not straightforward as there is still a lack of definitive surface markers for M1 macrophages and a lack of markers’ specificity for M2 macrophages [4,8,9]. It appears that the distinction between M1 and M2 may not be strictly relevant and that the overall degree of TAM infiltration within the tumor might be more important; accumulating evidence is suggesting overlapping features that depend on the mix of signals in their direct microenvironment [10,11]. 

The interest in TAMs has been also increasing in veterinary oncology where studies have mainly focused on mammary carcinomas and on colorectal carcinoma [12,13,14,15,16,17]. 

The aim of this study was to characterize the presence of TAMs in an heterogenous population of canine soft tissue sarcomas (STSs) and to analyze their relationship with tumor morphological features. For the purpose of this study, TAMs were identified as Iba-1 positive, non-neoplastic, tumor infiltrating cells [16]. The whole TAM population was analyzed, without differentiating between M1 and M2. We hypothesized that there would be an association between the presence of TAMs and tumor grading.

## 2. Materials and Methods

### 2.1. Samples

Pathology records of canine STSs submitted to the Section of Veterinary Pathology of the Department of Veterinary Anatomy, Physiology and Pathology of Liverpool University (UK), between January 2016 to December 2019, were searched in a computerized database. STSs excised for diagnostic and/or therapeutic purposes were included in the dataset. Cases were considered eligible for the study only if formalin fixed paraffin-embedded (FFPE) tissues were available for review and if neoplastic cells stained positive for vimentin, and negative for the calcium-binding adapter molecule 1 (Iba-1), Von Willebrand factor, and pan-cytokeratin.

Carcinosarcomas, oral and visceral STSs, as well as mesenchymal tumors such as synovial cell sarcoma, tumors of histiocytic origin, haemangiosarcoma, and undifferentiated tumors were excluded. 

Patient’s signalment and clinical data were retrieved from the submission forms/patient files; dogs were excluded from the study if they were known to have received steroids, non-steroidal anti-inflammatory drugs, and/or antineoplastic chemotherapy within a month prior to the tissue collection.

### 2.2. Histopathology and Immunohistochemistry

All histological slides were reviewed by a board-certified veterinary pathologist (LR). Slides were given a consecutive number so that the pathologist was blinded to the original pathology report and tumor/patient data (e.g., first or second opinion, tumor subtype and grade patient history, signalment, among others). 

The slides were originally prepared from FFPE tissues fixed in 10% neutral buffered formalin, were routinely stained with hematoxylin and eosin (HE), and were observed under a bright field upright microscope. Samples were assessed and graded following previous recommendations for the diagnosis of canine STS [18]. Briefly, assessed tumor areas were not overly complicated by inflammation and/or hemorrhage, as if so they were deselected. Differentiation represented the true differentiation of the tumor—uncertainty regarding histogenesis had no bearing on degree of differentiation [18]; sarcomas resembling adult normal tissue were given Score 1, sarcomas where the cell type could be determined but differentiation was poor were given Score 2, and sarcomas with a high degree of atypia (e.g., anisokaryosis and anisocytosis) where the origin was not determinable were given Score 3. Mitotic index (MI) was assessed within the most cellular part of the tumor and the area with the highest mitotic activity. The MI was calculated as the total number of mitotic figures in 10 tumor (microscopic 400×; ocular FN: 22; objective 40×/0.65) high-power fields (HPFs). According to STS grade, the number of mitoses in 10 high power fields was semi-quantitatively assessed using three scores: 0–9 mitoses (Score1), 10–19 mitoses (Score 2), >19 mitoses (Score 3). Necrosis was evaluated over the whole tumor sample present on the section, based on the % of area occupied by the necrotic process. A score was given as follows: no necrosis (Score 0), <50% of the tumor area as necrotic (Score 1), >50% of the tumor area as necrotic (Score 2). 

All the scores obtained from the three different assessments (differentiation, mitoses, necrosis) were summed, and Grade was determined as follows: Grade 1 (Score < 3); Grade 2 (Score 4 or 5), Grade 3 (Score 6 or above) [18].

Representative sections of the lesions were selected for immunohistochemistry (IHC). All sections were deparaffinized in xylene and hydrated with graded ethanol concentration up to distilled water. Antigen retrieval was performed by calibrated water bath capable of maintaining the epitope retrieval solution in 10 mM sodium citrate buffer (pH 6.0) at 97 °C for 30 min (Agilent technologies Ltd., Stockport, UK). The sections were allowed to cool down to room temperature for 20 min. Endogenous peroxidase was blocked using 100 µL Dako REAL^TM^ peroxidase blocking solution for 10 min (Agilent technologies Ltd., Stockport, UK). The following primary antibodies were selected: vimentin (Dako, mouse monoclonal, clone V9), Iba1 (Abcam, goat polyclonal, AB5076), Von Willebrand factor (Dako, rabbit polyclonal, A0082), and pan-cytokeratin (AE1/AE3/PCK26, Mouse monoclonal, Ready to use, Ventana). The bound antibody was evaluated by peroxidase conjugated polymers (Anti Mouse/Rabbit Envision Flex+, Agilent Technologies Ltd.; Agilent technologies Ltd., Stockport, UK or ImmPress anti Goat, Vector Laboratories, Burlingame, CA, USA) for 30 min and diaminobenzidine tetrahydrochloride was used as a detection system (DAB—Agilent Technologies Ltd., Stockport, UK). Upon completion of the immunostaining, sections were counterstained with Mayer’s hematoxylin. A normal canine lymph node was used as a positive control for Iba1 (nodal macrophages), vimentin (nodal stroma), and Von Willebrand factor (endothelium of the nodal vessels) antibodies; a section of normal skin was used as a positive control for pan-cytokeratin (epidermis). The negative control consisted of the substitution of the primary antibody with isotype-matched murine immunoglobulin or normal serum from rabbit/goat, depending on the primary antibody isotype. Tumor infiltrating, non-neoplastic, Iba-1 positive cells were considered as TAMs. 

Statistical association between number of TAMs and mitoses, necrosis scores, and tumor grade, was investigated using the Mann–Whitney test. Statistical analysis was performed using SPSS 13 Software (SPSS 13.0, SPSS Inc, IBM, Chicago, IL, USA). 

## 3. Results

Cases studied were gathered from 19 male and 19 female dogs, with a median age of 114.5 months (range: 12–168). There were 12 cross-breeds, 3 boxers, 3 Labrador retrievers, 2 border collies, 2 Doberman pinchers, 2 French bulldogs, and one each of the following breeds: bulldog, bull terrier, Cavalier King Charles spaniel, cocker spaniel, flat coated retriever, golden retriever, Jack Russel terrier, miniature schnauzer, Patterdale terrier, pit bull, Romanian shepherd, English setter, springer spaniel, and Staffordshire bull terrier. The locations of the neoplasms were as follows: limbs (17), trunk (15), and head and neck (6). Seven STSs were Grade 1, 13 were Grade 2, and 18 were Grade 3 (Table 1).

**Figure 1:**
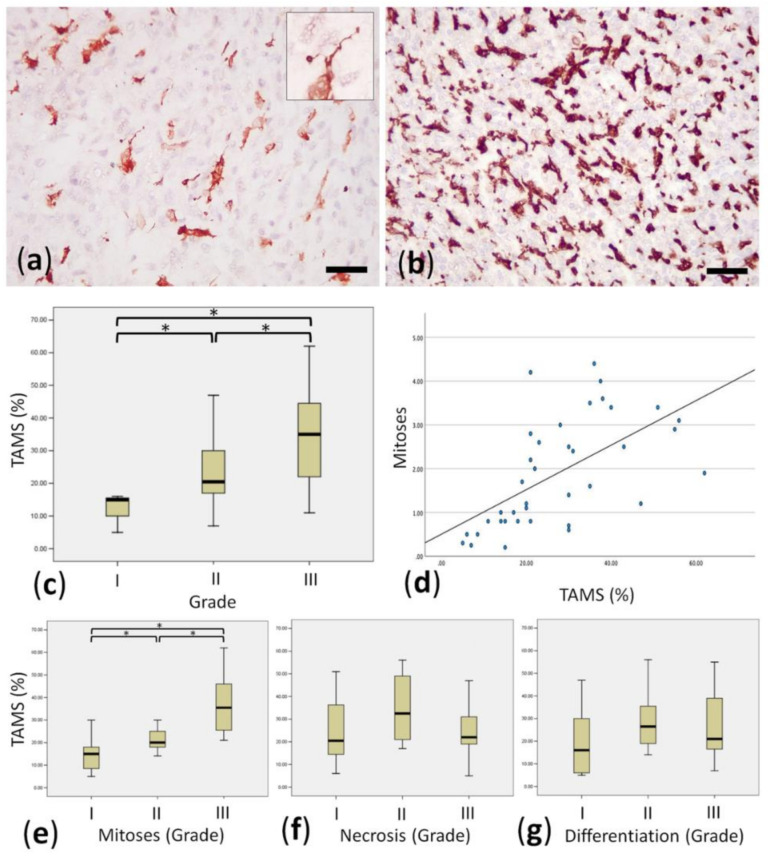
Morphological features of TAMs in canine soft tissue sarcoma, and their associations with grade. (**a**) Iba1-positive TAMs in a case with moderate TAM infiltration. Inset: often TAM exhibited dendritic-like cytoplasmic elongations. Scale bar: 50 microns, indirect immunoperoxidase. (**b**) Iba1-positive TAMs in a case with marked TAM infiltration. Scale bar: 50 microns; indirect immunoperoxidase. (**c**) Significant difference (“*”) in the median of % of TAM infiltration in different Grades. Scale bar: 50 microns; indirect immunoperoxidase. (**d**) Linear correlation between the average number of mitoses in 10 high-power fields (HPFs) and % of TAM infiltration. (**e**) Significant difference (“*”) in the median of % of TAM infiltration in different Grades considering only mitoses as grade component. (**f**) Lack of significant difference in the median of % of TAM infiltration in different Grades considering only necrosis as grade component. (**g**) Lack of significant difference in the median of % of TAM infiltration in different Grades considering only differentiation as grade component.

Iba1 immunohistochemistry successfully stained TAMs infiltrating within the STSs studied; these exhibited a predominant pleomorphic morphology with several dendritic-like cytoplasmic elongation, which embraced neoplastic cells (Figure 1a,b). 

The amount of TAMs, evaluated by the average percentage of Iba1 positive areas, varied widely between the tumours (median 21%; range 5–62%). When the percentage of positive TAMs of the STSs studied was associated with grade, a statistical association was detected (*p* < 0.05; Figure 1c). 

Investigating the possible association with TAM infiltration and each of the components of the STS grade (mitoses, differentiation, and percentage of necrosis), only the mitotic index was found to be positively associated with the three grade categories (Figure 1c,e–g). There was a linear association between mitoses and the percentage of the area occupied by TAMs (Figure 1d). No associations were found between TAMs and the age, sex, or tumour location.

## 4. Discussion

In the present study TAMs were investigated in canine STSs with the aid of Iba1 immunohistochemistry. The morphology of the cells was predominantly pleomorphic as expected from histiocytic cells, with occasional slender elongations, which suggest a contact with tumor cells. The most relevant finding of the present study is the association between the area occupied by TAMs and mitotic activity of the tumor. After unbundling the different descriptor of STS grading (differentiation, mitoses, necrosis), mitotic activity was the one only factor associated with TAMs. This may agree with the suggestion of some authors that TAMs are a prognostic indicator, and the higher the levels within the tumor the poorer the prognosis [19].

It is a common notion that TAMs are involved in the progression of tumors, however, the mechanism for tumor recruitment and the functions that allow these cells to help the tumor progressing are not fully understood [19]. Raposo et al. (2014) discuss in their article that TAMs have an important role in angiogenesis helping the sarcoma to proliferate. Similarly, TAMs are being reported to support carcinogenesis and tumor progression, which would concur with the findings of this study that the more TAMs present the higher the level of mitotic figures in tumor cells [13]. Similar findings have been reported by Valković et al. in human invasive ductal breast cancer, who noted that neoplastic lesions with a great number of TAMs had significantly higher mitotic activity than tumors with no or just a small focus of macrophages [20].

Conversely, we also consider alternative hypotheses for the correlation between the two factors. It could be possible that high levels of TAMs are attracted by the proliferating cells and proportional to their number in attempt to mitigate such proliferation or without any biological further action. Since no functional studies are available that investigate the insights of these mechanisms, this remains an association to further explore. The results of this study do not clarify a positive or negative mechanistic effect of TAMs in canine soft tissue sarcomas.

Using TAMs as a therapeutic target is a well discussed topic. TAMs stimulate angiogenesis as a response to hypoxic conditions, which are in part a result of cancer therapies; therefore, having a potential negative effect on therapeutic attempts [13]. Targeting these macrophages might lead to better attempts at therapy as these cells would no longer be “defending” the tumor. Some authors suggest that directly targeting TAMs is in itself cancer therapy and new ways of carrying this out are being currently investigated [19]. Franklin et al. noted that when certain signaling proteins are prevented, the differentiation of TAMs is blocked. This depletion then led to a reduced metastatic rate and increased the potential of T cytotoxic lymphocytes to suppresses tumor growth [21]. More recent human studies have clarified the heterogeneity and complexity of the immune microenvironment and the pervasive immunosuppressive niche sustained by TAMs, which may be overcome with the activation of T cells that traffic into STSs [22]. Overall, it appears that therapeutic success may be significantly improved by the development of a more effective combination of chemo-immunotherapies in STSs [23].

A still complex topic is to clarify if a difference exists between tissue resident macrophages that happen to infiltrate the tumor in a site, or distant recruited macrophages that infiltrate a specific tumor site. Researchers such as Franklin et al. and Wu et al. suggest that tissue resident macrophages have little effect on tumor burden and overall malignancy, and that some even suppress tumor advancement [21,24]. An example is microglia in the brain, which according to Sarkar et al. suppresses brain tumor initiating cells during tumorigenesis or relapse of gliomas [25]. In contrast to this, Wu et al. describe how resident Kupffer cells in the liver can be shown to promote the progression of hepatocarcinoma [24]. This is still a complex field and more research needs to be conducted to ensure the correct molecular characterization of macrophages to determine whether they are TAMs recruited at distance or resident tissue associated macrophages [26]. In our study we have not differentiated between resident and distantly recruited macrophages, but we have instead considered the whole population of TAMs as per pattern of infiltration and Iba1 positivity [16]. 

While TAMs are at the forefront of many investigations into metastatic tumors, research focusing on soft tissue sarcomas is limited. There are human models based on lung cancer and mammary tumors in canines, of which principles are transferable [13,14,27]. However, it should be noted that there is a vast array of research on this matter in human patients, but limited information is available in the veterinary field. More research in the veterinary field may find different results than those that are presented in the human field as in previous cases. 

A limitation of the present study is the lack of further characterization of TAMs into M1 and M2 subtypes; however, the liner correlation observed between iba1 positive cells and mitotic activity (and ultimately grading) represents a relevant finding. 

## 5. Conclusions

Results of this study suggest that that distinction between M1 and M2 might be more important than the overall presence of TAMs within the tumor, as supported by other authors [10,11]. It is the opinion of the authors that STSs with high number of TAMs may pose a significant challenge to pathologists, with the potential of being misdiagnosed for histiocytic sarcomas, and caution should be made during interpretation of HE and IHC slides. Further studies are warranted to investigate the biological mechanisms of TAMs in canine sarcomas. 

## Figures and Tables

**Table 1:** Characteristics of the study population. Signalment, anatomic STS location, and histologic grade.

Breed	Age (Months)	Sex	STS Anatomical Location	STS Grade
Doberman pincher	64	Female	Limb	1
Miniature schnauzer	72	Female	Head and Neck	1
Border collie	120	Male	Limb	1
Cavalier King Charles spaniel	120	Male	Limb	1
Cross-breed	110	Female	Trunk	1
Labrador retriever	120	Female	Trunk	1
Border collie	129	Male	Limb	1
Doberman pincher	12	Female	Limb	2
Labrador retriever	57	Male	Head and Neck	2
Cross-breed	120	Male	Trunk	2
Cross-breed	132	Female	Limb	2
Cross-breed	120	Female	Trunk	2
Cross-breed	143	Male	Head and Neck	2
Boxer	146	Female	Limb	2
Cross-breed	120	Female	Limb	2
Cross-breed	96	Female	Limb	2
Boxer	114	Female	Limb	2
French bulldog	84	Male	Head and Neck	2
Cross-breed	120	Female	Limb	2
Staffordshire bull terrier	96	Male	Limb	2
Cocker spaniel	132	Male	Trunk	3
Cross-breed	84	Male	Trunk	3
Bulldog	60	Male	Trunk	3
Boxer	24	Female	Trunk	3
Bull terrier	132	Male	Limb	3
Springer spaniel	168	Male	Trunk	3
English setter	115	Female	Limb	3
French bulldog	96	Male	Head and Neck	3
Patterdale terrier	144	Female	Head and Neck	3
Jack Russel terrier	115	Male	Trunk	3
Romanian shepherd	36	Female	Limb	3
Golden retriever	81	Female	Trunk	3
Flat-coated retriever	84	Female	Trunk	3
Labrador retriever	144	Male	Trunk	3
Pit bull	76	Male	Trunk	3
Cross-breed	132	Male	Limb	3
Cross-breed	100	Female	Limb	3
Cross-breed	96	Male	Trunk	3

## Data Availability

Data are available upon reasonable request.
